# Anti-inflammatory and arthritic effects of thiacremonone, a novel sulfurcompound isolated from garlic via inhibition of NF-κB

**DOI:** 10.1186/ar2819

**Published:** 2009-09-30

**Authors:** Jung Ok Ban, Ju Hoon Oh, Tae Myoung Kim, Dae Joong Kim, Heon-Sang Jeong, Sang Bae Han, Jin Tae Hong

**Affiliations:** 1 College of Pharmacy and Medical Research Center, Chungbuk National University, 12, Gaeshin-dong, Heungduk-gu, Cheongju, Chungbuk, 361-763, Korea; 2 College of Veterinary Medicine, Chungbuk National University, 12, Gaeshin-dong, Heungduk-gu, Cheongju, Chungbuk, 361-763, Korea; 3 College of Agriculture, Life and Environments Sciences, Chungbuk National University, 12, Gaeshin-dong, Heungduk-gu, Cheongju, Chungbuk, 361-763, Korea

## Abstract

**Introduction:**

Sulfur compounds isolated from garlic exert anti-inflammatory properties. We recently isolated thiacremonone, a novel sulfur compound from garlic. Here, we investigated the anti-inflammatory and arthritis properties of thiacremonone through inhibition of NF-κB since NF-κB is known to be a target molecule of sulfur compounds and an implicated transcription factor regulating inflammatory response genes.

**Methods:**

The anti-inflammatory and arthritis effects of thiacremone in *in vivo *were investigated in 12-O-tetradecanoylphorbol-13-acetate-induced ear edema, carrageenan and mycobacterium butyricum-induced inflammatory and arthritis models. Lipopolysaccharide-induced nitric oxide (NO) production was determined by Griess method. The DNA binding activity of NF-κB was investigated by electrophoretic mobility shift assay. NF-κB and inducible nitric oxide synthetase (iNOS) transcriptional activity was determined by luciferase assay. Expression of iNOS and cyclooxygenase-2 (COX-2) was determined by western blot.

**Results:**

The results showed that topical application of thiacremonone (1 or 2 μg/ear) suppressed the 12-O-tetradecanoylphorbol-13-acetate-induced (1 μg/ear) ear edema. Thiacremonone (1-10 mg/kg) administered directly into the plantar surface of hind paw also suppressed the carrageenan (1.5 mg/paw) and mycobacterium butyricum (2 mg/paw)-induced inflammatory and arthritic responses as well as expression of iNOS and COX-2, in addition to NF-κB DNA-binding activity. In further in vitro study, thiacremonone (2.5-10 μg/ml) inhibited lipopolysaccharide (LPS, 1 μg/ml)-induced nitric oxide (NO) production, and NF-κB transcriptional and DNA binding activity in a dose dependent manner. The inhibition of NO by thiacremonone was consistent with the inhibitory effect on LPS-induced inducible nitric oxide synthase (iNOS) and COX-2 expression, as well as iNOS transcriptional activity. Moreover, thiacremonone inhibited LPS-induced p50 and p65 nuclear translocation, resulting in an inhibition of the DNA binding activity of the NF-κB. These inhibitory effects on NF-κB activity and NO generation were suppressed by reducing agents dithiothreitol (DTT) and glutathione, and were abrogated in p50 (C62S)-mutant cells, suggesting that the sulfhydryl group of NF-κB molecules may be a target of thiacremonone.

**Conclusions:**

The present results suggested that thiacremonone exerted its anti-inflammatory and anti-arthritic properties through the inhibition of NF-κB activation via interaction with the sulfhydryl group of NF-κB molecules, and thus could be a useful agent for the treatment of inflammatory and arthritic diseases.

## Introduction

Garlic has been used in traditional medicine as a food component to prevent the development of cancer and cardiovascular diseases, by modifying risk factors such as hypertension, high blood cholesterol and thrombosis, and preventing other chronic diseases associated with aging [1-4]. These pharmacological effects of garlic are attributed to the presence of pharmacologically active sulfur compounds including diallyl sulfide, diallyl disulfide, allicin, and dipropyl sulfide. These compounds have been known to increase the activity of enzymes involved in the metabolism of carcinogens [5], and have anti-oxidative activities [6] as well as anti-inflammatory effects *in vitro *and *in vivo *[7-13]. Despite their widespread medicinal use and anti-inflammatory effects, little is known about the cellular and molecular mechanisms of the components of garlic.

Nuclear factor (NF)-κB is a family of transcription factors that includes RelA (p65), NF-κB1 (p50 and p105), NF-κB2 (p52 and p100), c-Rel, and RelB. These transcription factors are sequestered in the cytoplasm by inhibitory (I) κBs, which prevent NF-κB activation, and inhibit nuclear accumulation. The degradation of IκBs facilitates the migration of NF-κB into the nucleus, where they typically form homodimers or heterodimers that bind to the promoters of many inflammatory response genes and activate transcription [14,15]. Targeted disruption of the p50 subunit of NF-κB reduces ventricular rupture as well as improving cardiac function and survival after myocardial infarction, a proinflammatory disease [16,17]. It is also well appreciated that p50 homodimers are important in the inflammatory cytokine genes, and that the ratio of p50 relative to the other Rel (p65) family members in the nucleus is likely to be a determining factor for gene expression of inflammation. NF-κB regulates host inflammatory and immune response properties by increasing the expression of specific cellular genes [18]. These include the transcription of various inflammatory cytokines, such as IL-1, IL-2, IL-6, IL-8 and TNF-α [19], as well as genes encoding cyclooxygenase-2 (COX-2) and iNOS. As a result, inhibition of signal pathways leading to inactivation of NF-κB is now widely recognized as a valid strategy combating autoimmune, inflammatory, and osteolytic diseases [20].

Several studies have shown that inhibitors of NF-κB may be useful in the treatment of inflammatory diseases including arthritis [21-23]. Anti-inflammatory drugs have also been demonstrated to inhibit the NF-κB pathway [24-26]. We recently also found that inhibition of NF-κB can ameliorate inflammatory responses, and arthritis [27-30]. Several recent investigations have shown that sulfur compounds can effectively interfere with the NF-κB pathway [31-33]. In a series of pharmacological studies of sulfur compound in garlic, we found that the antioxidant properties of garlic-water extract is increased by a raise in the heating temperature of the extract. We isolated and identified thiacremonone, a novel and major sulfur compund (0.3%) in garlic, and found that it has higher anti-oxidant properties compared with other sulfur compounds [34,35]. We also reported an inhibitory effect of thiacremonone on NF-κB activity in colon carcinoma cell lines, in parallel with the inhibitory effect of colon cell growth and induction of apoptosis [15]. In this study, we investigated whether thiacremonone exerted anti-inflammatory and arthritis effects through the inhibition of NF-κB activity.

## Materials and methods

### Chemicals

**Figure 1:**
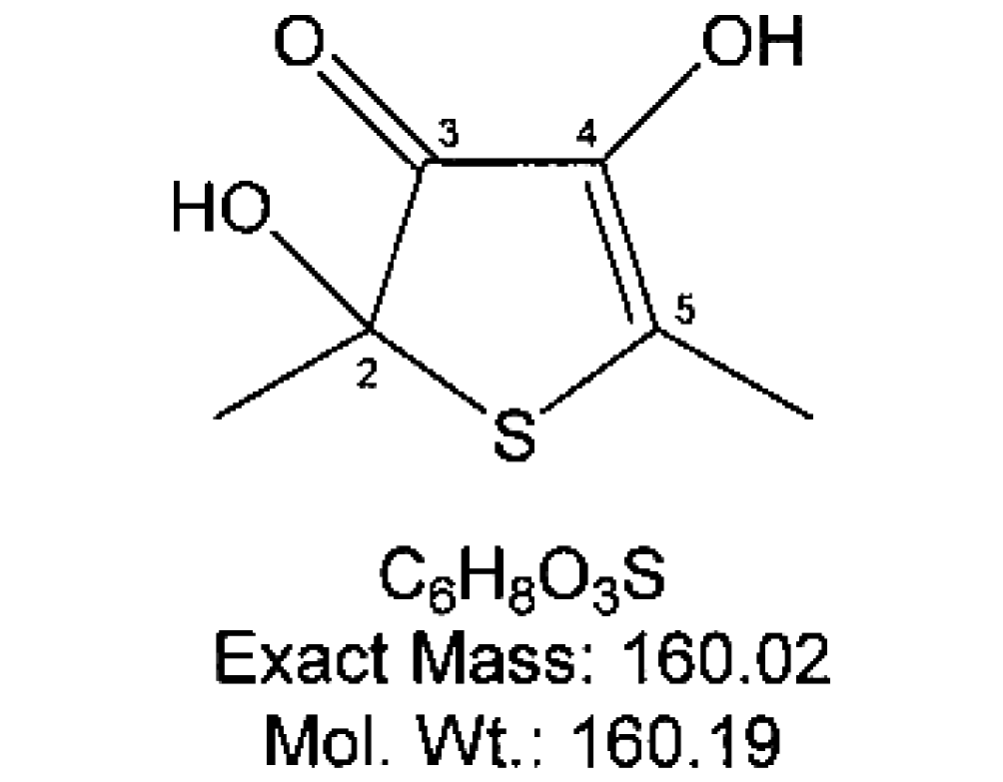
Chemical structure of thiacremonone.

Characterization of a novel sulfur compound isolated from garlic (named thiacremonone) has been described elsewhere [15,34]. Its structure is shown in Figure 1. Thiacremonone was resolved in 0.01% dimethyl sulfoxide, and treated at sample sizes of 2.5, 5 and 10 μg/ml in culture cells.

### Cell culture

RAW 264.7, a mouse macrophage-like cell line and THP-1, a human monocytic cell line, were obtained from the American Type Culture Collection (Cryosite, Lane Cove, NSW, Australia). DMEM, RPMI, penicillin, streptomycin, and fetal bovine serum were purchased from Gibco Life Technologies (Rockville, MD, USA). RAW 264.7 cells were grown in DMEM with 10% fetal bovine serum, 100 U/ml penicillin, and 100 μg/ml streptomycin at 37°C in 5% carbon dioxide (CO_2_) humidified air. THP-1 cells were grown in RPMI with 10% fetal bovine serum, 0.05 mM 2-mercaptoethanol, 100 U/ml penicillin, and 100 μg/ml streptomycin at 37°C in 5% CO_2 _humidified air.

### Cell viability assay

RAW 264.7 cells were plated at a density of 10^4 ^cells/well in 96-well plates. To determine the appropriate dose that is not cytotoxic to the cells, the cytotoxic effect was evaluated in the cells cultured for 24 hours using the cell counting kit-8 assay according to the manufacturer's instructions (Dojindo, Gaithersburg, MD, USA). Briefly, 10 μl of the cell counting kit-8 (CCK-8) solution was added to cell culture, and incubated for a further 24 hours. The resulting color was assayed at 450 nM using a microplate absorbance reader (Sunrise, Tecan, Switzerland). Each assay was carried out in triplicate.

### Nitrite assay

RAW 264.7 cells were plated at 2 × 10^4 ^cells/well in 96-well plate and then incubated with or without lipopolysaccharide (LPS; 1 μg/ml) in the absence or presence of various concentrations of thiacremonone for 24 hours. The nitrite accumulation in the supernatant was assessed by Griess reaction [36]. Each 50 μl of culture supernatant was mixed with an equal volume of Griess reagent (0.1% *N*-(1-naphthyl)-ethylenediamine, 1% sulfanilamide in 5% phophoric acid) and incubated at room temperature for 10 minutes. The absorbance at 550 nm was measured in an automated microplate reader, and a series of known concentrations of sodium nitrite was used as a standard.

### Electromobility shift assay

Electromobility shift assay (EMSA) was performed as described previously [15]. Briefly, 1 × 10^6 ^cells/ml was washed twice with 1 × PBS, followed by the addition of 1 ml of PBS, and the cells were scraped into a cold Eppendorf tube. Cells were spun down at 15,000 *g *for one minutes, and the resulting supernatant was removed. Solution A (50 mM HEPES, pH 7.4, 10 mM KCl, 1 mM EDTA, 1 mM EGTA, 1 mM dithiothreitol, 0.1 μg/ml phenylmethylsulfonyl fluoride, 1 μg/ml pepstatin A, 1 μg/ml leupeptin, 10 μg/ml soybean trypsin inhibitor, 10 μg/ml aprotinin, and 0.5% Nonidet P-40) was added to the pellet in a 2:1 ratio (v/v) and incubated on ice for 10 minutes. Solution C (solution A + 10% glycerol and 400 mM KCl) was added to the pellet in a 2:1 ratio (v/v) and vortexed on ice for 20 minutes. The cells were centrifuged at 15,000 *g *for seven minutes, and the resulting nuclear extract supernatant was collected in a chilled Eppendorf tube. Consensus oligonucleotides were end-labeled using T4 polynucleotide kinase and (γ -^32^P) ATP for 10 minutes at 37°C. Gel shift reactions were assembled and allowed to incubate at room temperature for 10 minutes followed by the addition of 1 μl (50,000 to 200,000 cpm) of ^32^P-labeled oligonucleotide and another 20 minutes of incubation at room temperature. Subsequently 1 μl of gel loading buffer was added to each reaction and loaded onto a 4% nondenaturing gel and electrophoresed until the dye was 75% of the way down the gel. The gel was dried at 80°C for one hour and exposed to film overnight at 70°C. The relative density of the protein bands was scanned by densitometry using MyImage (SLB, Seoul, Korea), and quantified by Labworks 4.0 software (UVP Inc., Upland, CA, USA). The relative density of the DNA-protein binding bands was scanned by densitometry using MyImage (SLB, Seoul, Korea), and quantified by Labworks 4.0 software (UVP Inc, Upland, CA, USA).

### Transfection and assay of luciferase activity

RAW 264.7 cells (5 × 10^6 ^cells) were plated in 24-well plates and transiently transfected with pNF-κB-Luc plasmid (5 × NF-κB; Stratagene, La Jolla, CA, USA) or iNOS-luciferase reporter plasmid [37] or p50 (C62S) mutant plasmids using a mixture of plasmid and lipofectAMINE PLUS in OPTI-MEN according to manufacturer's specification (Invitrogen, Carlsbad, CA, USA). Cells were transiently co-transfected with pEGFP-C1 vector (Clontech, Palo Alto, CA, USA) with WelFect-EX™ *PLUS *transfection reagent (WelGENE Inc., Daegu, Korea) according to the manufacturer's instructions. After 24 hours transfection, expression of green fluorescent protein (GFP) was detected by fluorescence microscopy (DAS microscope: Leica Microsystems, Inc., Deefield, IL, USA). The transfection efficiency was determined as the number of GFP-expressing cells divided by the total cell number counted × 100.

The transfected cells were treated with LPS (1 μg/ml) and different concentrations (2.5, 5 and 10 μg/ml) of thiacremonone for eight hours. Luciferase activity was measured by using the luciferase assay kit (Promega, Madison, WI, USA), and reading the results on a luminometer as described by the manufacturer's specifications (WinGlow, Bad Wildbad, Germany).

### Western blot analysis

Western blot analysis was performed as described previously [15]. The membrane was incubated for five hours at room temperature with specific antibodies: mouse polyclonal antibodies against p50 and p-IκB (1:500 dilution, Santa Cruz Biotechnology Inc. Santa Cruz, CA, USA), rabbit polyclonal for p65 and IκB (1:500 dilution, Santa Cruz Biotechnology Inc., Santa Cruz, CA, USA) and iNOS and COX-2 (1:1000 dilution, Cayman Chemical, Ann Arbor, MI, USA). The blot was then incubated with the corresponding conjugated anti-mouse immunoglobulin G-horseradish peroxidase (1:4,000 dilution, Santa Cruz Biotechnology Inc., Santa Cruz, CA, USA). Immunoreactive proteins were detected with the enhanced chemiluminescence (ECL) western blotting detection system (GE Healthcare Biosciences (formerly Amersham Biosciences), Little Chalfont, Buckinghamshire, UK). The relative density of the protein bands was scanned by densitometry using MyImage (SLB, Seoul, Korea), and quantified by Labworks 4.0 software (UVP Inc., Upland, CA, USA).

### Assay of 12-O-tetradecanoylphorbol-13-acetate-induced ear edema in mice

The male Institute of Cancer Research (ICR) mice and male Sprague-Dawley (SD) rats used here were maintained in accordance with the National Institute of Toxicological Research of the Korea Food and Drug Administration guidelines for the care and use of laboratory animals. The protocol was approved by the Institutional Animal Care and Use Committee at Chungbuk National University. 12-O-tetradecanoylphorbol-13-acetate (TPA; 1 μg/ear) alone or in combination with thiacremonone (1 or 2 μg/ear) in acetone (10 μl) was applied to the right ear of ICR mice. Control mice received acetone alone. A volume (10 μL) of thiacremonone (1 or 2 μg/ear) containing acetone was delivered to both the inner and outer surfaces of the ear 30 minutes after TPA application. After 24 hours, the tip of the ear thickness was measured using vernier calipers (Mitutoyo Corporation, Kawasaki, Japan), and ear punch biopsies 6 mm in diameter were taken and weighed. Following this, the mice were sacrificed by cervical dislocation. The increase in thickness or weight of the ear punches was directly proportional to the degree of inflammation [38]. We further investigated the expression of iNOS and COX-2 by western blot analysis, and the activation of NF-κB by EMSA in each ear punch biopsies.

### Carrageenan-induced paw edema inflammatory model and *Mycobacterium butyricum*-induced arthritis model

The anti-inflammatory and anti-arthritic property of thiacremonone was tested in male SD rats using the carrageenan paw edema test according to the method of Sugishita and colleagues [39] and a *Mycobacterium butyricum*-induced arthritic model as described elsewhere [27]. Thiacremonone (1 or 2 mg/kg), indomethacin (positive control, 10 mg/kg) or vehicle (saline) was administered directly into the plantar surface of the right hind paw 30 minutes after injection of carrageenan (0.05 ml; 3%, w/v in saline) into the subplantar area of the right hind paw. The volumes of the injected and contralateral paws were measured at one, two, three, and four hours after induction of edema using a plethysmometer (Letica, Comella, Spain). We next investigated the antiarthritic effect of thiacremonone in a chronic adjuvant-induced arthritis (AIA) animal model. AIA was elicited in SD rats by the injection of 0.1 ml of *M. butyricum *(10 mg/ml) in saline, into the subplantar area of the right hind paw. Paw volumes were measured at the beginning of the experiment using a water-displacement plethysmometer. Animals with edema values of 1.1 ml larger than normal paws were then randomized into treatment groups. A 10 mg/kg dose of thiacremonone, indomethacin (positive control) or vehicle (saline) was subcutaneously administered into the plantar surface of the right hind paw from day 1 to day 20 post AIA induction. The magnitude of the inflammatory response was evaluated by measuring the volumes of both hind paws. On day 21 post AIA induction, rats under anesthesia were placed on a radiographic box at a distance of 90 cm from an x-ray source. Radiographic analysis of arthritic hind paws was performed using an x-ray machine (BLD-150RK, Hradec Králové, Czech Republic) with a 40 KW exposition for 0.01 seconds. Paws were oriented horizontally, relative to the detector. Radiographs were scored by an investigator who was blinded to the treatment information, using the following scale: 0 = no bone damage, 1 = tissue swelling and edema, 2 = joint erosion, and 3 = bone erosion and osteophyte formation.

### Data analysis

Data were analyzed using one-way analysis of variance followed by Tuckey test as a *post hoc *test. Differences were considered significant at *P *< 0.05.

## Results

### Inhibitory effect of thiacremonone on TPA-induced ear edema in mice

**Figure 2:**
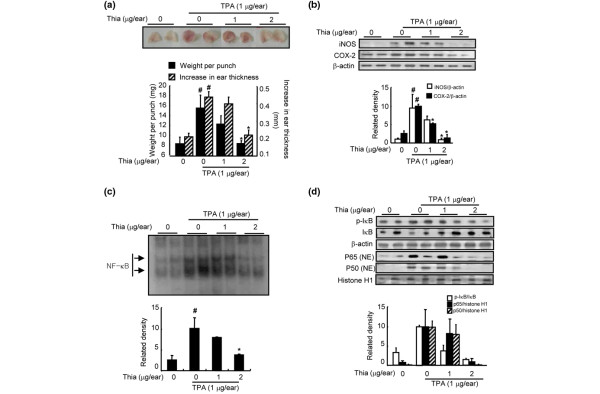
Effects of thiacremonone on TPA-induced ear edema, and expression of iNOS and COX-2 in mice.  **(a) **12-O-tetradecanoylphorbol-13-acetate (TPA; 1 μg/ear) alone or together with thiacremonone (Thia; 1 or 2 μg/ear) in 10 μl acetone was topically applied to the right ear of Institute of Cancer Research (ICR) mice (n = 6). The thickness or weight of the ear punches were determined as described in Materials and Methods. **(b) **Equal amounts of total proteins (40 μg/lane) were subjected to 10% SDS-PAGE, and the expression of inducible nitric oxide synthetase (iNOS) and cyclooxygenase-2 (COX-2) in mice ear edema tissues (2 lanes/each group) was detected by western blotting using specific antibodies. β-actin protein was used as an internal control. **(c) **DNA-binding activity of nuclear factor (NF)-κB was determined by electromobility shift assay (EMSA) in nuclear extracts from mice ear edema tissues (2 lanes/each group) as described in Materials and Methods. **(d) **Equal amounts of total proteins (40 μg/lane) were subjected to 10% SDS-PAGE, and nuclear translocation of p50 and p65, and degradation of inhibitory (I) κB in mice ear edema tissues (2 lanes/each group) was detected by western blotting using specific antibodies. β-actin protein was used as an internal control. Values are mean ± standard deviation (n = 6). ^# ^indicates significantly different from control group (*P *< 0.05). * *P *< 0.05 indicate statistically significant differences from the TPA-treated group.

Thiacremonone was evaluated for its anti-inflammatory activity against TPA-induced edema formation and inflammatory gene expression as well as NF-κB activity in mice. Topical application of 1 μg TPA in acetone to the ear of a mouse increased the average weight of the ear from 4.3 mg to 7.2 mg at 24 hours post application (Figure 2a). Topical application of 1 or 2 μg thiacremonone together with 1 μg TPA to the ears of mice inhibited the TPA-induced edema of mouse ears by 44 or 98%, respectively (Figure 2a). We further investigated the effect of thiacremonone on iNOS and COX-2 expression and NF-κB activity in each ear punch biopsies by western blot analysis and EMSA. Thiacremonone dose-dependently inhibited TPA-induced expression of iNOS and COX-2 (Figure 2b). Thiacremonone also inhibited TPA-induced NF-κB DNA-binding activity (Figure 2c) as well as the nuclear translocation of p50 and p65 and phosphorylation of IκBα (Figure 2d).

### Inhibitory effect of thiacremonone on carrageenan and adjuvant-induced arthritis

**Figure 3:**
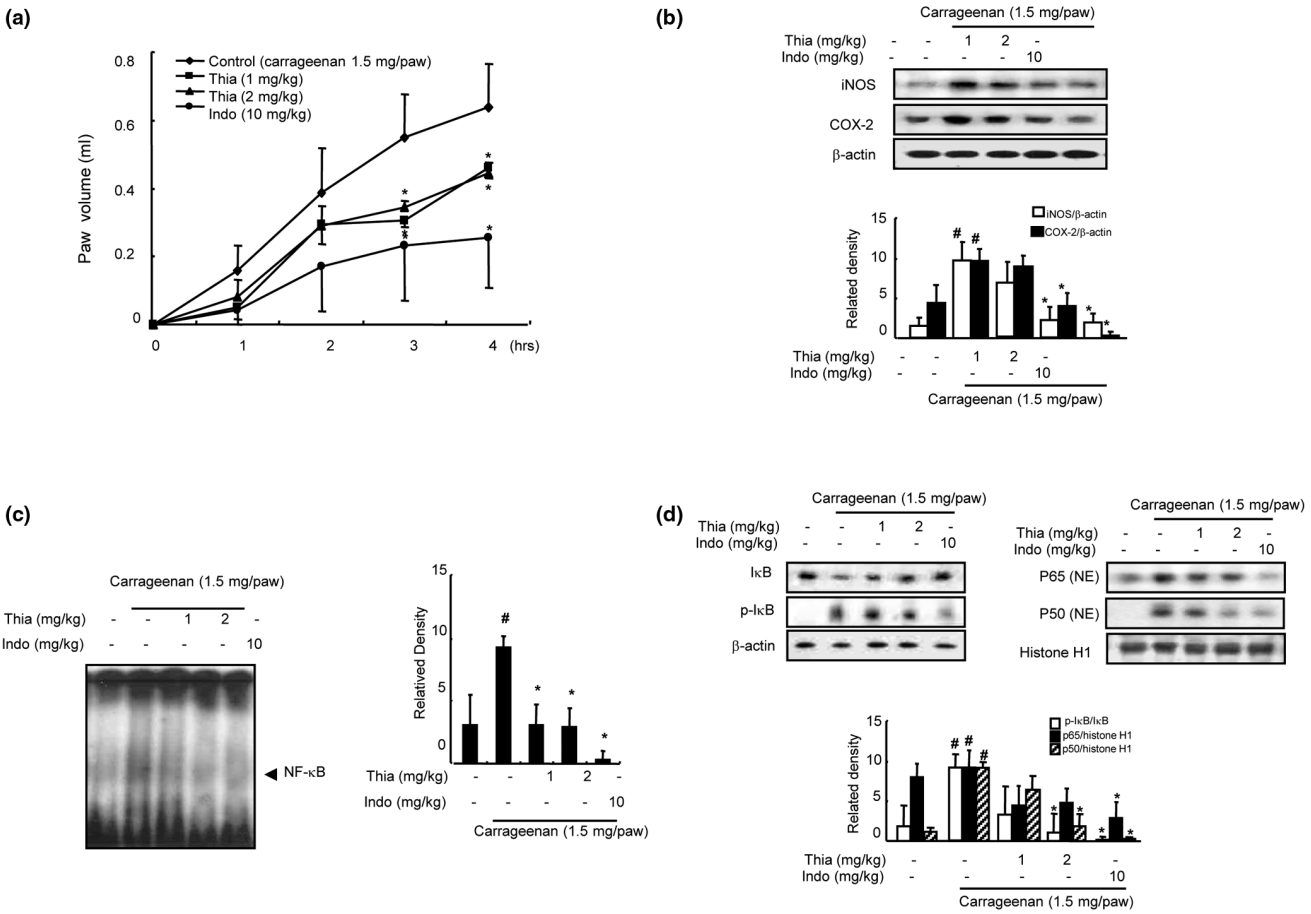
Effect of thiacremonone on carrageenan-induced arthritis in rats.  **(a) **Thiacremonone (Thia; 1 and 2 mg/kg) or indomethacin (Indo; 10 mg/kg) or vehicle (saline) was orally administered 30 minutes before carrageenan (0.05 ml; 3%, w/v in saline) into the planter area of the right hind paw of rat (n = 10). The volumes of the injected paws were monitored for four hours in 10 rats per each group as described in Materials and Methods. **(b) **Equal amounts of total proteins (40 μg/lane) were subjected to 10% SDS-PAGE, and the expression of inducible nitric oxide synthetase (iNOS) and cyclooxygenase-2 (COX-2) in rat paw arthritis tissues was detected by western blotting using specific antibodies. β-actin protein was used as an internal control. **(c) **DNA-binding activity of nuclear factor (NF)-κB was determined by electromobility shift assay (EMSA) in nuclear extracts from mice paw arthritis tissues (3 lanes/each group) as described in Materials and Methods. **(d) **Equal amounts of total proteins (40 μg/lane) were subjected to 10% SDS-PAGE, and nuclear translocation of p50 and p65, and degradation of inhibitory (I) κB in rat paw arthritis tissues was detected by western blotting using specific antibodies. β-actin protein was used as an internal control. Values are mean ± standard deviation (n = 10). ^# ^indicates significantly different from control group (*P *< 0.05). * *P *< 0.05 indicate statistically significant differences from the carrageenan-treated group.

**Figure 4:**
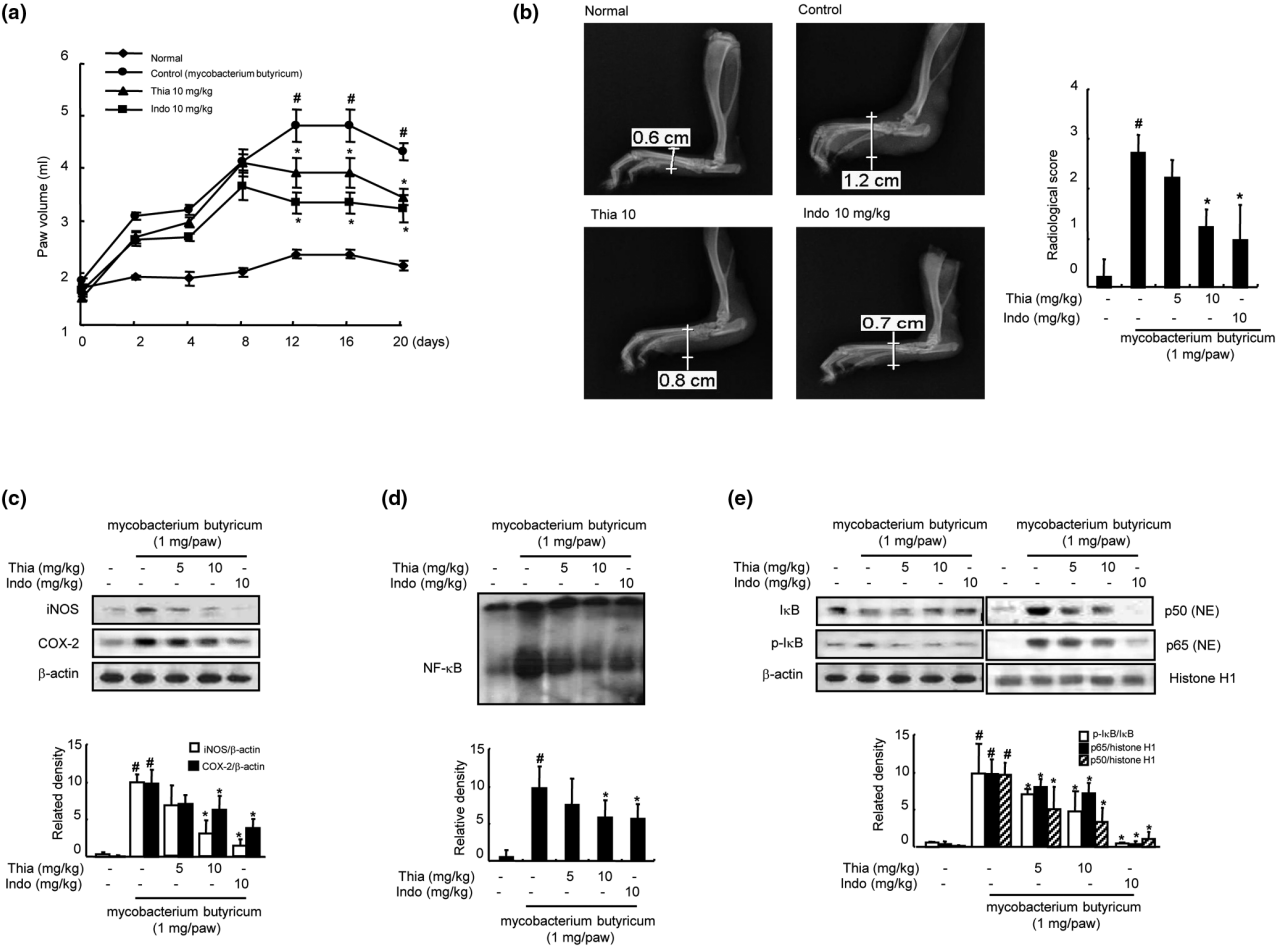
Effect of thiacremonone on adjuvant-induced arthritis in rats.  **(a) **Thiacremonone (Thia; 10 mg/kg) and indomethacin (Indo; 10 mg/kg) were orally administered for 20 days after injection of adjuvant into the plantar surface of right hind paw of 10 rats per group. Hind paw volume and clinical score were determined for 20 days as described in Materials and Methods. **(b) **A radiographic examination of hind paws revealed tissue swelling at the paw after 20 days. The clinical value was determined in 10 rats as described in Materials and Methods. **(c) **Equal amounts of total proteins (40 μg/lane) were subjected to 10% SDS-PAGE, and the expression of inducible nitric oxide synthetase (iNOS) and cyclooxygenase-2 (COX-2) in rat paw arthritis tissues (3 lanes/each group) was detected by western blotting using specific antibodies. β-actin protein was used as an internal control. **(d) **DNA-binding activity of nuclear factor (NF)-κB was determined by electromobility shift assay (EMSA) in nucleus extract from rat paw arthritis tissues (3 lanes/each group) as described in Materials and Methods. **(e) **Equal amounts of total proteins (40 μg/lane) were subjected to 10% SDS-PAGE, and nuclear translocation of p50 and p65, and degradation of inhibitory (I) κB in rat paw arthritis tissues was detected by western blotting using specific antibodies. β-actin protein was used as an internal control. Values are mean ± standard deviation (n = 10). ^# ^indicates significantly different from control group (*P *< 0.05). * indicates significantly different from the *Mycobacterium butyricum*-treated group (*P *< 0.05).

The anti-inflammatory activity of thiacremonone was also demonstrated in the carrageenan paw edema test in SD rats. Direct administration of thiacremonone (1 or 2 mg/kg) into the plantar surface of the right hind paw 30 minutes before injection of carrageenan (0.05 ml; 3%, w/v in saline into the subplantar area of the right hind paw, 1.5 mg/paw) showed greatly reduced carrageenan-induced paw edema (40% reduction compared to contralateral paws; Figure 3a). A dose-dependent inhibition of the expression of iNOS and COX-2 (Figure 3b) as well as the activation of NF-κB DNA-binding activity (Figure 3c) accompanied by an inhibition of p50 and p65 nuclear translocation and phosphorylation of IκBα (Figure 3d) was also reported. In a chronic rat AIA model, oral administration of thiacremonone (5 or 10 mg/kg) for 20 days significantly reduced adjuvant-induced hind paw edema formation (Figure 4a). A radiographic examination of hind paws revealed tissue swelling at the paw of adjuvant-injected rats. However, these effects were markedly reduced by thiacremonone treatment, and its inhibitory effect was comparable with indomethacin (10 mg/kg; Figure 4b). Treatment with thiacremonone did not affect progression of body weight, and did not show any behavioral alternation (data not shown), suggesting that thiacremonone itself (10 mg/kg) did not cause any toxic response. Thiacremonone dose-dependently inhibited the expression of iNOS and COX-2 (Figure 4c). It also suppressed the activation of NF-κB DNA-binding activity (Figure 4d) as well as the nuclear translocation of p50 and p65 and phosphorylation of IκBα (Figure 4e).

### Effect of thiacremonone on NF-κB-luciferase activity and NF-κB DNA binding activity

**Figure 5:**
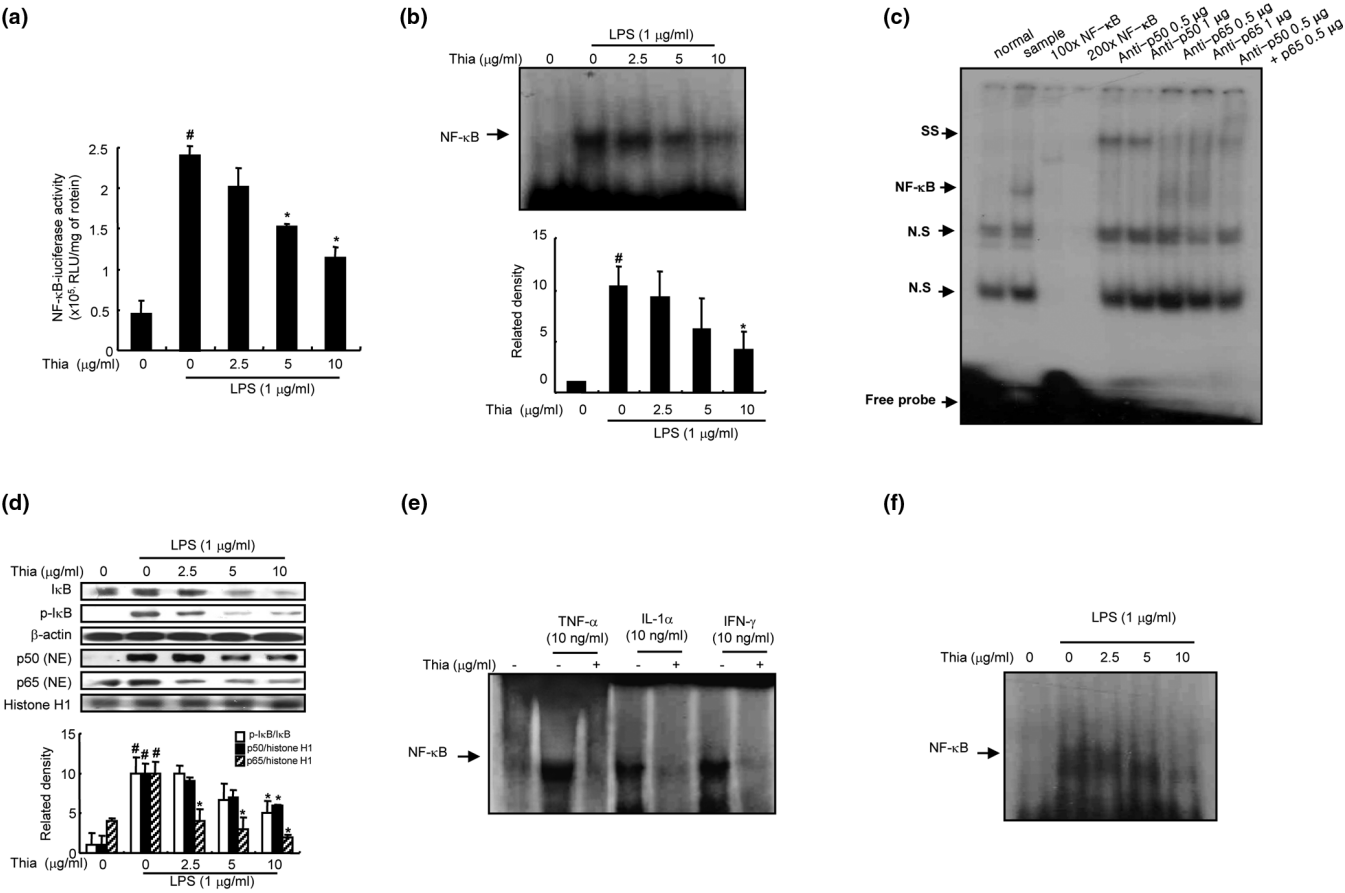
Effect of thiacremonone on LPS-induced NF-κB activation in RAW 264.7 and THP-1 cells.  **(a) **RAW 264.7 cells were transfected with p-NF-κB-Luc plasmid (5× nuclear factor (NF)-κB), and then treated with lipopolysaccharide (LPS; 1 μg/ml) alone or in combination with thiacremonone (Thia; 2.5, 5, 10 μg/ml) at 37°C for six hours. Luciferase activity was then determined as described in Materials and Methods. **(b) **The DNA-binding activity of NF-κB was investigated using electromobility shift assay (EMSA) as described in Materials and Methods. Nuclear extracts from RAW 264.7 cells with LPS alone (1 μg/mL) or in combination with thiacremonone (2.5, 5, 10 μg/ml) were subjected to DNA-binding reactions with ^32^P end-labeled oligonucleotide specific to NF-κB. The specific DNA-binding activity of NF-κB complex is indicated by an arrow. **(c) **For competition assays, nuclear extracts from RAW 264.7 cells treated with LPS (1 μg/ml) were incubated for one hour before EMSA with unlabeled NF-κB oligonucleotide or labeled NF-κB oligonucleotide. For supershift assays, nuclear extracts from RAW 264.7 cells treated with LPS (1 μg/ml) were incubated for one hour before EMSA with specific antibodies against the p50 and p65 NF-κB isoforms. SS indicates supershift band. **(d) **Cells treated with 1 μg/mL of LPS only or LPS plus different concentrations (2.5, 5, 10 μg/ml) of thiacremonone at 37°C for one hour. Equal amounts of total protein (40 μg) were subjected to 10% SDS-PAGE. Nuclear translocation of p50 and p65, and degradation of inhibitory (I) κB were detected by western blotting using specific antibodies. β-actin protein was used as an internal control. **(e) **Nuclear extracts from RAW 264.7 cells with another inducer alone (TNF-α (10 ng/ml), IL-1α (10 ng/ml), IFN-γ (10 ng/ml)) or in combination with thiacremonone (10 μg/ml) were subjected to DNA-binding reactions with ^32^P end-labeled oligonucleotide specific to NF-κB. The specific DNA-binding of NF-κB complex is indicated by an arrow. **(f) **Nuclear extracts from THP-1 cells with LPS alone (1 μg/mL) or in combination with thiacremonone (2.5, 5, 10 μg/ml) were subjected to DNA-binding reactions with ^32^P end-labeled oligonucleotide specific to NF-κB. The specific DNA-binding of NF-κB complex is indicated by an arrow. Values (A, B and C) are mean ± standard deviation of three independent experiments performed in triplicate. ^# ^indicates significantly different from control group (*P *< 0.05). * indicates significantly different from the LPS-treated group (*P *< 0.05).

To test whether thiacremonone was able to attenuate NF-κB-mediated promoter activity, we used a luciferase reporter gene expressed under the control five κB *cis*-acting elements. RAW 264.7 cells were transiently transfected with an NF-κB-dependent luciferase reporter construct according to the manufacturef's specifications (Promega, Madison, WI, USA). The cells were then treated with LPS (1 μg/ml) or co-treated with LPS and thiacremonone for six hours. Treatment of cells with thiacremonone resulted in a concentration-dependent suppression of luciferase activity induced by LPS (Figure 5a). To determine whether thiacremonone was also able to inhibit the DNA-binding activity of NF-κB in RAW 264.7 cells, nuclear extracts from co-treated cells were prepared and assayed for NF-κB DNA-binding activity by EMSA. LPS induced a strong NF-κB DNA-binding activity that was attenuated by co-treatment of the cells with thiacremonone in a dose-dependent manner (Figure 5b).

Treatment of cells with LPS (1 μg/ml) increased the nuclear translocation of NF-κB subunits p65 and p50. However, in the presence of thiacremonone, nuclear translocation of p50 and p65 was inhibited in a dose-dependent manner (Figure 4c). Thiacremonone also inhibited LPS-induced degradation of IκB-α (increase phosphorylation) in RAW 264.7 cells (Figure 5c). We also found that exposure of RAW 264.7 cells to thiacremonone for one hour inhibited the DNA-binding activity of NF-κB that was induced by TNF-α (10 ng/ml), IL-1α (10 ng/ml) and interferon-γ (IFN-γ; 10 ng/ml; Figure 5d). The dose-dependent inhibitory effect of thiacremonone on LPS-induced DNA binding activity of NF-κB was also seen in THP-1 cells (Figure 5e). This DNA-binding activity of NF-κB was confirmed by competition assays as well as by super shift assays. In the presence of a p50 antibody, the DNA-binding activities of NF-κB showed a super shift. However, in the presence of a p65 antibody, the DNA-binding activity of NF-κB was decreased without a super shift, suggesting that p50 might be a target of thiacremonone, interfering with the DNA-binding activity of NF-κB (Figure 5c).

### Effect of thiacremonone on LPS-induced NO production as well as expression of iNOS and COX-2 in RAW 264.7 cells

**Figure 6:**
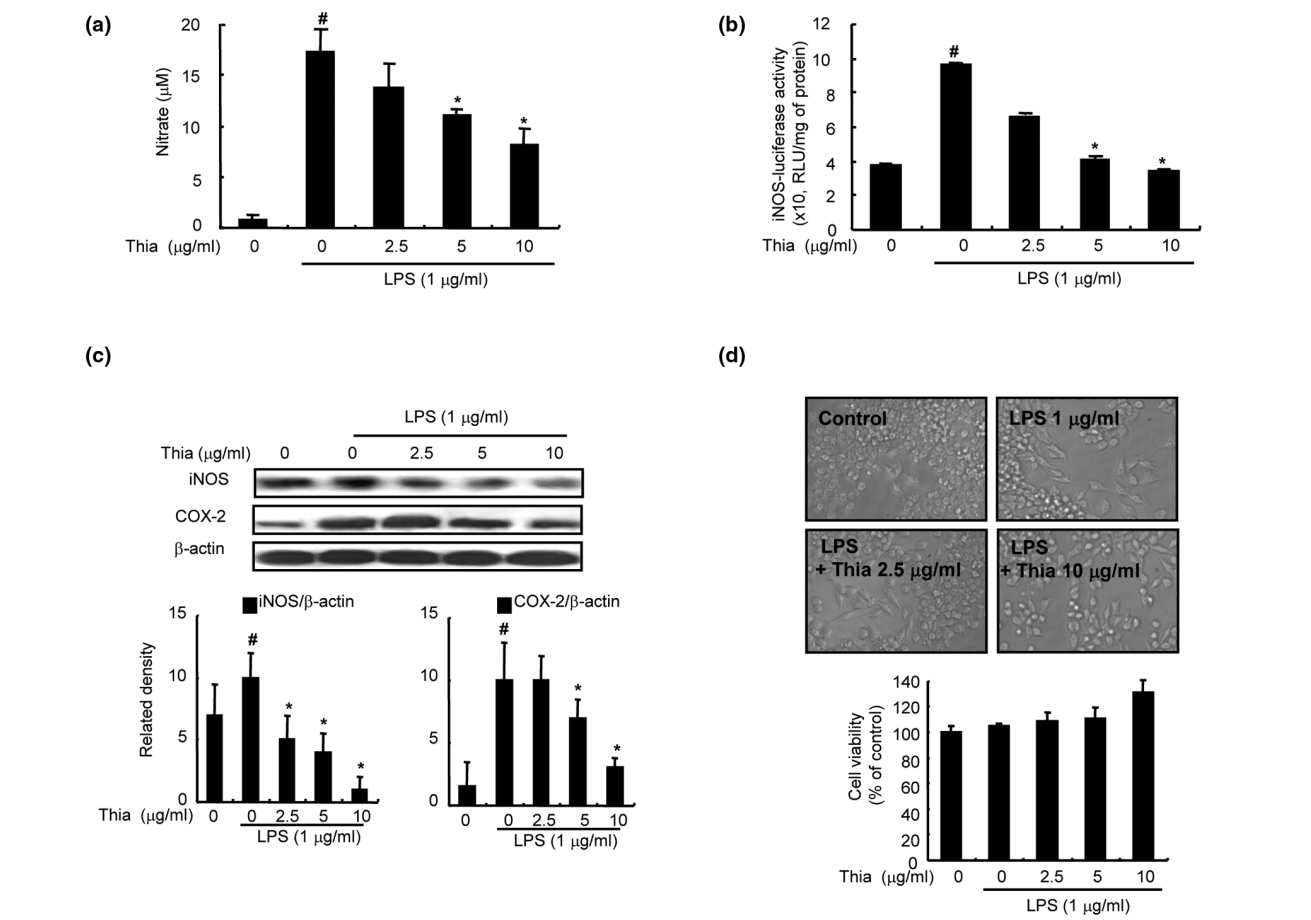
Effect of thiacremonone on LPS-induced NO generation, expression of iNOS and COX-2 and cell viability in RAW 264.7 cells.  **(a) **The cells were treated with 1 μg/mL of lipopolysaccharide (LPS) only or LPS combined with different concentrations (2.5, 5, 10 μg/ml) of thiacremonone (Thia) at 37°C for 24 hours. Nitric oxide (NO) generation was determined in culture medium as described in Materials and Methods. **(b) **The cells were transiently transfected with an inducible nitric oxide synthetase (iNOS)-luciferase construct, and activated with LPS (1 μg/ml) alone or LPS combined with the indicated concentrations of thiacremonone for eight hours. Luciferase activity was then determined. Quantification of band intensities from three independent experimental results was determined by a densitometry, and the value under the band indicate fold difference (average) from untreated control group. **(c) **The cells were treated with 1 μg/mL of LPS only or LPS combined with different concentrations (2.5, 5, 10 μg/ml) of thiacremonone at 37°C for 24 hours. Equal amounts of total proteins (40 μg/lane) were subjected to 10% SDS-PAGE, and the expression of iNOS and COX-2 was detected by western blotting using specific antibodies. β-actin protein was used as an internal control. **(d) **RAW 264.7 cells were treated with various doses (2.5, 5, 10 μg/ml) of thiacremonone for 24 hours. Morphological changes were observed under microscope (magnification, ×200). Cell viability was determined by the CCK-8 assay described in Materials and Methods. Cells were incubated with thiacremonone in the absence or presence of LPS. Results were given in percentage related to untreated controls. All values (A, B, C and D) represent the means ± standard deviation of three independent experiments performed in triplicate. ^# ^indicates significantly different from control group (*P *< 0.05). * indicates significantly different from the LPS-treated group (*P *< 0.05).

The effect of thiacremonone (2.5, 5, 10 μg/ml) on LPS-induced NO production in RAW 264.7 cells was investigated by measuring the accumulated nitrite, as estimated by Griess reaction, in the culture medium. After co-treatment with LPS and thiacremonone for 24 hours, LPS-induced nitrite concentration in the medium was decreased remarkably in a concentration-dependent manner. The IC_50 _value of thiacremonone in inhibiting LPS-induced NO production was 8 μM (Figure 6a).

To investigate whether the inhibitory effect of thiacremonone affected NO production via inhibition of corresponding gene expression, iNOS luciferase activity and expression of iNOS and COX-2 was determined. Transcriptional regulation of iNOS expression by thiacremonone was determined in RAW 264.7 transfected with iNOS-luciferase construct containing murine iNOS promoter (-1592/+183) fused to luciferase gene as a reporter [39]. Thiacremonone inhibited LPS-induced iNOS luciferase activity in a concentration-dependent manner (Figure 6b). Upon LPS treatment for 24 hours, iNOS expression was also significantly increased in RAW 264.7 cells, and co-treatment of cells with LPS and different concentration of thiacremonone decreased LPS-induced iNOS expression in a concentration-dependent manner (Figure 6c). In agreement with the inhibitory effect on NO generation, the densitometry data showed that the iNOS expression was inhibited by thiacremonone in a concentration-dependent manner. As NO can induce COX-2 expression, and COX-2 is also an enzyme to regulate inflammation, the expression of COX-2 was investigated. Consistent with the inhibitory effect on iNOS expression, thiacremonone inhibited LPS-induced COX-2 expression, but the extent was much less than on iNOS (Figure 6c).

To disprove the inhibitory effect of thiacremonone on NO production via inhibition of cell growth, the cytotoxic effect of thiacremonone was evaluated in the absence or presence of LPS in the RAW 264.7 cells by CCK-8 assay. Thiacremonone (up to 10 μg/ml) did not affect the cell viability in the absence of LPS (data not shown) or the presence of LPS in RAW 264.7 cells (Figure 6d). Therefore, thiacremonone inhibited LPS-induced NO production in RAW 264.7 cells without any toxic effect.

### Suppression of thiacremonone-induced inhibition of DNA binding activity of NF-κB and cell growth by thiol reducing agents, and in the cells transfected with mutant p50

**Figure 7:**
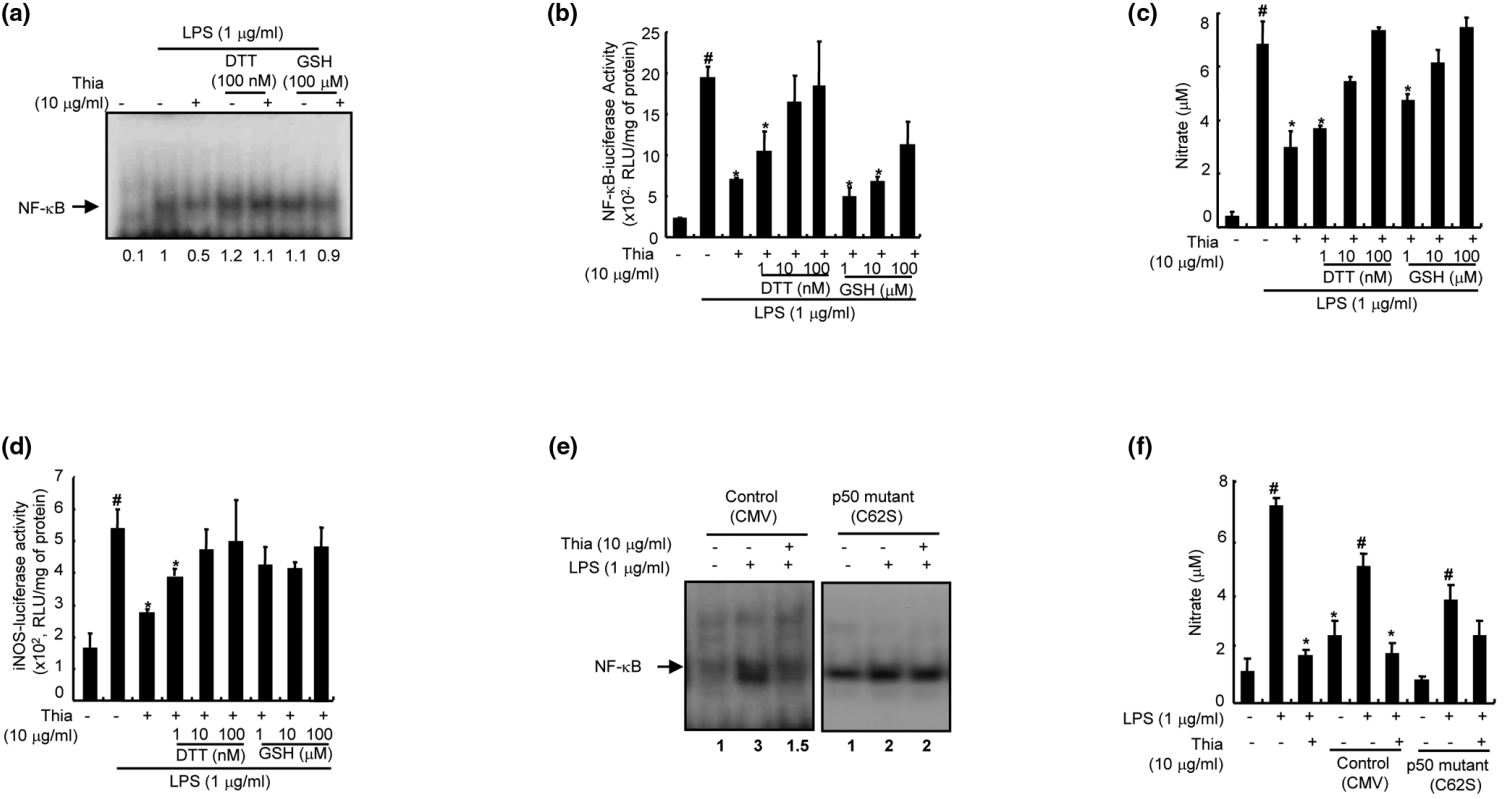
Abolition of the inhibitory effect of thiacremonone by DTT and glutathione GSH, and in the cells harboring mutant p50 on NO generation and DNA binding activation of NF-κB.  **(a) **RAW 264.7 cells grown in six-well plates were cotreated with indicated concentrations of dithiothreitol (DTT) (100 nM) or glutathione (GSH; 100 μM) with thiacremonone (Thia; 10 μg/ml) for one hour. Nuclear extracts were then prepared and examined by electromobility shift assay (EMSA) as described in Materials and Methods. **(b) **The cells were transiently transfected with nuclear factor (NF)-κB-luciferase construct, and were co-treated with indicated concentrations of DTT (1 to 100 nM) or GSH (1 to 100 μM) with thiacremonone (10 μg/ml) for eight hours, and then the luciferase activity was determined. **(c) **The cells were co-treated with indicated concentrations of DTT (1 to 100 nM) or GSH (1 to 100 μM) with 1 μg/mL of lipopolysaccharide (LPS) only or LPS plus thiacremonone (10 μg/ml) at 37°C for 24 hours. Nitric oxide (NO) generation was determined in culture medium as described in Materials and Methods. **(d) **The cells were transiently transfected with inducible nitric oxide synthetase (iNOS)-luciferase construct, and were co-treated with indicated concentrations of DTT (1 to 100 nM) or GHS (1 to 100 μM) with thiacremonone (10 μg/ml) for eight hours, and then the luciferase activity was determined. **(e) **RAW 264.7 cells were transfected with p50 mutant (C62S) plasmid at 37°C for six hours, and then NF-κB DNA-binding activity was determined after one hour of treatment with thiacremonone by electromobility shift assay (EMSA) as described in Materials and Methods. **(f) **NO generation was determined in culture medium as described in Materials and Methods. RAW 264.7 cells were transfected with p50 mutant (C62S) plasmid at 37°C for six hours, and then NO generation was determined after 24 hours treatment with thiacremonone as described in Materials and Methods. All values represent the means ± standard deviation of three independent experiments performed in triplicate. ^# ^indicates significantly different from control group (*P *< 0.05). * *P *< 0.05 indicate statistically significant differences from the LPS-treated group.

We further tested whether the inhibition of NF-κB was due to an interaction between the sulfhydryl group of the p50 subunit of NF-κB and thiacremonone, as previously seen in colon cancer cells [15]. Cells were co-treated with thiacremonone and reducing agents, dithiothreitol (DTT) or glutathione for one hour, and then the DNA-binding activity of NF-κB was examined. We found that these reducing agents significantly suppressed the inhibitory effects of thiacremonone on the DNA-binding and transcriptional activity of NF-κB (Figures 7a, b). Furthermore, DTT and glutathione suppressed the inhibitory effects of thiacremonone on NO generation (Figure 7c) and iNOS luciferase activity (Figure 7d).

Taking into consideration the supershift of the DNA-binding activities of NF-κB upon addition of anti-p50 antibody, and the suppressive effect of DTT and glutathione on thiacremonone-induced inhibition of DNA-binding activity of NF-κB and NO generation, we postulated that the sulfhydryl residue in p50 might be a target of thiacremonone. To test this postulation, we further studied the inhibitory effects of thiacremonone on the DNA-binding activity of NF-κB and NO generation in p50 mutant cells (C62S), where the cysteine residue at 62 of p50 was replaced by serine. As expected, there was a reduction in the inhibitory effect of thiacremonone on the DNA-binding activity of NF-κB (Figure 7e) and on NO generation (Figure 7f) in these p50 mutant cells. These results clearly suggested that thiacremonone mediated its effects through modulation of cysteine residues of the p50 subunit of NF-κB.

## Discussion

The activation of iNOS catalyzes the formation of a large amount of NO, which plays a key role in the pathogenesis of a variety of inflammatory diseases [40-43]. Activation of NF-κB is critical in the induction of iNOS [44-46]. Therefore, agents that inhibit NF-κB, resulting in decreased iNOS expression and NO generation, may have beneficial therapeutic effects in the treatment of inflammatory diseases. Thiacremonone inhibited LPS-induced iNOS and COX-2 expression accompanied by a reduction in NO generation. Consistent with its inhibitory activity on NO production, thiacremonone also decreased NF-κB activity. The inhibitory effects of thiacremonone on the NF-κB DNA-binding activities were also demonstrated in macrophages stimulated by TNF-α, IFN-γ, and IL-1α. The promoter of the iNOS gene contains two major discrete regions synergistically functioning toward the binding of transcription factor NF-κB, which is mainly activated by LPS and IFN-γ, and IL-1α [47,48]. Therefore, these data indicated that thiacremonone could interfere with NF-κB-mediated signals involving the production of pro-inflammatory molecule NO, and thus give anti-inflammatory responses.

*In vivo *animal studies showed that thiacremonone inhibited TPA, carrageenan and *M. butyricum*-induced paw edema. Treatment of thiacremonone also resulted in a great reduction of tissue swelling and osteophyte formation in a chronic arthritis rat model. Paralleled with these inhibitory effects, thiacremonone also inhibited TPA, carrageenan and *M. butyricum*-induced iNOS and COX-2 expression, as well as NF-κB activity *in vivo*. Thiacremonone inhibited the production of TNF-α as well as the expression of matrix metalloproteinases (MMP-3 and 9) and chemokines in these tissues (data not shown). Activation of the NF-κB pathway results in the transactivation of a multitude of responsive genes that contribute toward the inflammatory phenotype, including TNF-α from macrophages, MMPs from synovial fibroblasts and chemokines that recruit immune cells to the inflamed pannus. This is largely a consequence of the activation of the NF-κB pathway that involves homodimers and heterodimers of p50/p65 [49]. We thus speculated that the *in vivo *effects of thiacremonone on arthritic models were mediated by its combined inhibitory actions on multiple responses of synovial cells and inflammatory cells through the inactivation of NF-κB. Interestingly; we also found that thiacremonone inhibited NF-κB and iNOS expression in cultured THP-1 monocytes. In light of these data, the results of our study indicate that inhibition of NF-κB by thiacremonone could be beneficial for the treatment of inflammatory diseases such as arthritis.

The inhibition of NF-κB activation by thiacremonone was found to be suppressed by treatment of cells with reducing agents such as DTT and glutathione. This was accompanied by a suppression of the inhibitory effect of thiacremonone on NO generation. Thus, it is possible that the inhibitory effects of thiacremonone on NF-κB activity may be mediated by oxidizing the critical cysteine residue present in NF-κB subunits. We also found that in the presence of an antibody against p50 but not p65, the NF-κB DNA-binding activity was supershifted. Further evidence showed that the inhibitory effect of NO generation and NF-κB activity by thiacremonone in p50 mutant (cysteine was replaced with alanine) cells was suppressed. It is noteworthy that p50/p50 homodimer is more important than p50/p65 heterodimers in the regulation of inflammatory cytokine generation and inflammatory diseases. It was found that increased cytokine levels in p50 knockout mice may be related to the different transcriptional activity of p50/p50 homodimer rather than p65/p50 heterodimer or p65/p65 homodimer [50]. Targeted disruption of the p50 subunit of NF-κB reduces atherosclerotic lesions with an inflammatory phenotype as well as ventricular rupture after myocardial infarction, a proinflammatory disease [51,52]. These results suggest that p50/p50 may be more important to relay inflammatory gene expression than that of p65/p50 or p65/65 in the inflammatory responses. Therefore, there studies support the possibility that the sulfhydryl residue of p50 may be a target of thiacremonone in the present study. Previous our study demonstrated that thiacremonone inhibited cancer cell growth through inhibition of NF-κB, and may be p65 is the target of thiacremonone [15]. Contrast to the inflammatory response, in the cancer cells, p65 may be important in the activation of NF-κB, and many of anti-cancer drugs target p65 of NF-κB. Our data in the cancer cell study is consistent with those previously reported from other laboratory with caffeic acid phenethyl ester [53] and sesquiterpene lactone parthenolide [54]. Several other investigators demonstrated that sulfur compounds react with cysteine residues of target molecules in intracellular signal transduction proteins including NF-κB through cysteine-cysteine interaction or other binding ways, and thus inhibit inflammatory responses and development of arthritic rheumatism [14,31,32]. We recently also demonstrated that 2-hydroxycinnamaldehyde, a snake venom toxin and melittin inhibit inflammatory responses and cancer cell growth through modification of sulfhydryl residues of NF-κB and regulatory proteins (p50 and p65 as well as IκB kinases (IKKs)) [27,28,55]. Therefore, the inhibition of NF-κB activation by thiacremonone through direct modification of p50 may be an important molecular mechanism of the suppressive effect of thiacremonone on inflammatory responses and arthritic reactions. However, in our present study, thiacremonone inhibited both the expression of IκB as well as its phosphorylation, but the extent of the inhibition of phosphorylation was much greater than the inhibition of IκB expression. Thus, these results could give possibilities that thiacremonone can suppress the expression of IκB and p-IκB as well as inhibit phosphorylation. As the sulfhydryl group of IKKs are also important in the activity of IKKs as well as NF-κB, thiacremonone could be effective in the regulation of IKKs. We are currently investigating these issues.

The effective dose of thiacremonone (10 mg/kg) used in this chronic AIA study was comparable with that of the classic anti-inflammatory drug indomethacin. We did not detect any side effects of thiacremonone (loss of weight gain and any observed toxic signs) during treatment for 20 days. Taken together, thiacremonone, a novel sulfur compound isolated from garlic inhibited iNOS expression and NO generation through prevention of NF-κB activity *in vitro*, and ameliorated inflammatory responses and arthritic reactions in acute and chronic edema and arthritic animal models. These data suggest that thiacremonone may be potentially beneficial for the prevention of inflammatory diseases such as arthritic rheumatism with comparatively low toxic effects.

## Conclusions

Our results indicate that thiacremonone suppressed the TPA-induced ear edema, and carrageenan and *M. butyricum*-induced arthritis through inhibition of NF-κB DNA-binding activity and expression of iNOS and COX-2. In *in vitro *studies using Raw 264.7 and THP-1 cells, thiacremonone also inhibited LPS-induced NO production, NF-κB activity and expression of iNOS and COX-2, which are classical markers of inflammation. These inhibitory effects were suppressed by reducing agents such as DTT and glutathione, and were abrogated in the cells expressing p50 (C62S) mutant. Therefore, we conclude that thiacremonone exerted its anti-inflammatory and anti-arthritic properties through the inhibition of NF-κB activation via interaction with the sulfhydryl group of NF-κB molecules.

## Abbreviations

AIA: adjuvant-induced arthritis; CCK-8: cell counting kit-8; CO_2_: carbon dioxide; COX-2: cyclooxygenase-2; DMEM: Dulbecco's modified eagle medium; DTT: dithiothreitol; ECL: enhanced chemiluminescence; EMSA: electromobility shift assay; GFP: green fluorescent protein; ICR: Institute of Cancer Research; IκB: inhibitory κB; IFN: interferon; IKK: inhibitory κB kinase; IL: interleukin; iNOS: inducible nitric oxide synthetase; LPS: lipopolysaccharide; MMP: matrix metalloproteinases; NF: nuclear factor; NO: nitric oxide; PBS: phosphate-buffered saline; SD: Sprague-Dawley; TNF: tumor necrosis factor; TPA: 12-O-tetradecanoylphorbol-13-acetate.

## Competing interests

The authors declare that they have no competing interests.

## Authors' contributions

JTH conceived the design of this study and coordinated all phases of the preparation of the manuscript. JOB, JHO and TMK performed the experiments, and JOB participated in the statistical analysis. DJK performed radiographic analysis of arthritic hind paws and HJ isolated thiacremonone from garlic and provided. SBH participated in data analysis and helped to draft the manuscript. All authors read and approved the final manuscript.

## References

[B1] Rahman K Historical perspective on garlic and cardiovascular disease J Nutr 2001 131 977S 979S 11238800 10.1093/jn/131.3.977S

[B2] Tanaka S Haruma K Yoshihara M Kajiyama G Kira K Amagase H Chayama K Aged garlic extract has potential suppressive effect on colorectal adenomas in humans J Nutr 2006 136 821S 826S 16484573 10.1093/jn/136.3.821S

[B3] Rahman K Garlic and aging: new insights into an old remedy Ageing Res Rev 2003 2 39 56 10.1016/S1568-1637(02)00049-1 12437995

[B4] Neil A Silagy C Garlic: its cardiovascular-protective properties Curr Opin Lipidol 1994 5 989S 993S 10.1097/00041433-199402000-00002 15559024

[B5] Fisher CD Augustine LM Maher JM Nelson DM Slitt AL Klaassen CD Lehman-McKeeman LD Cherrington NJ Induction of drug-metabolizing enzymes by garlic and allyl sulfide compounds via activation of car and CAR and Nrf2 Drug Metab Dispos 2007 35 995 1000 10.1124/dmd.106.014340 17353348

[B6] Pari L Murugavel P Sitasawad SL Kumar KS Cytoprotective and antioxidant role of diallyl tetrasulfide on cadmium induced renal injury: an in vivo and in vitro study Life Sci 2007 80 650 658 10.1016/j.lfs.2006.10.013 17125799

[B7] Narayanaswami V Sies H Antioxidant activity of ebselen and related selenoorganic compounds in microsomal lipid peroxidation Free Radic Res Commun 1990 10 237 244 10.3109/10715769009149892 2289694

[B8] Sabayan B Foroughinia F Chohedry A A postulated role of garlic organosulfur compounds in prevention of valproic acid hepatotoxicity Med Hypotheses 2007 68 512 514 10.1016/j.mehy.2006.07.055 17046167

[B9] Murugavel P Pari L Effects of diallyl tetrasulfide on cadmium-induced oxidative damage in the liver of rats Hum Exp Toxicol 2007 26 527 534 10.1177/0960327107073810 17698948

[B10] Park EY Ki SH Ko MS Kim CW Lee MH Lee YS Kim SG Garlic oil and DDB, comprised in a pharmaceutical composition for the treatment of patients with viral hepatitis, prevents acute liver injuries potentiated by glutathione deficiency in rats Chem Biol Interact 2005 155 82 96 10.1016/j.cbi.2005.04.006 15950962

[B11] Lang A Lahav M Sakhnini E Barshack I Fidder HH Avidan B Bardan E Hershkoviz R Bar-Meir S Chowers Y Allicin inhibits spontaneous and TNF-alpha induced secretion of proinflammatory cytokines and chemokines from intestinal epithelial cells Clin Nutr 2004 23 1199 1208 10.1016/j.clnu.2004.03.011 15380914

[B12] Son EW Mo SJ Rhee DK Pyo S Inhibition of ICAM-1 expression by garlic component, allicin, in gamma-irradiated human vascular endothelial cells via downregulation of the JNK signaling pathway Int Immunopharmacol 2006 6 1788 1795 10.1016/j.intimp.2006.07.021 17052669

[B13] Chiang YH Jen LN Su HY Lii CK Sheen LY Liu CT Effects of garlic oil and two of its major organosulfur compounds, diallyl disulfide and diallyl trisulfide, on intestinal damage in rats injected with endotoxin Toxicol Appl Pharmacol 2006 213 46 54 10.1016/j.taap.2005.08.008 16274720

[B14] Kim KM Chun SB Koo MS Choi WJ Kim TW Kwon YG Chung HT Billiar TR Kim YM Differential regulation of NO availability from macrophages and endothelial cells by the garlic component S-allyl cysteine Free Radic Biol Med 2001 30 747 756 10.1016/S0891-5849(01)00460-9 11275474

[B15] Ban JO Hwang IG Kim TM Hwang BY Lee US Jeong HS Yoon YW Kimz DJ Hong JT Inhibition of cell growth and induction of apoptosis via inactivation of NF-kappaB by a sulfurcompound isolated from garlic in human colon cancer cells J Pharmacol Sci 2007 104 374 383 10.1254/jphs.FP0070789 17721042

[B16] Häcker H Karin M Regulation and function of IKK and IKK-related kinases Sci STKE 2006 357 re13 10.1126/stke.3572006re13 17047224

[B17] Guha M Mackman N LPS induction of gene expression in human monocytes Cell Signal 2001 13 85 94 10.1016/S0898-6568(00)00149-2 11257452

[B18] Yamamoto Y Gaynor RB Therapeutic potential of inhibition of the NF-κB pathway in the treatment of inflammation and cancer J Clin Invest 2001 107 135 142 10.1172/JCI11914 11160126 PMC199180

[B19] Haefner B NF-κB: arresting a major culprit in cancer Drug Discov Today 2002 7 653 663 10.1016/S1359-6446(02)02309-7 12110242

[B20] Abu-Amer Y Darwech I Otero J Role of the NF-kappaB axis in immune modulation of osteoclasts and bone loss Autoimmunity 2008 41 204 211 10.1080/08916930701694543 18365833

[B21] Lu JW Wang H Yan-Li J Zhang C Ning H Li XY Zhang H Duan ZH Zhao L Wei W Xu DX Differential effects of pyrrolidine dithiocarbamate on TNF-alpha-mediated liver injury in two different models of fulminant hepatitis J Hepatol 2008 48 442 452 10.1016/j.jhep.2007.10.014 18215436

[B22] Okamoto H Iwamoto T Kotake S Momohara S Yamanaka H Kamatani N Inhibition of NF-kappaB signaling by fenofibrate, a peroxisome proliferator-activated receptor-alpha ligand, presents a therapeutic strategy for rheumatoid arthritis Clin Exp Rheumatol 2005 23 323 330 15971419

[B23] Stio M Martinesi M Bruni S Treves C Mathieu C Verstuyf A d'Albasio G Bagnoli S Bonanomi AG The Vitamin D analogue TX 527 blocks NF-kappaB activation in peripheral blood mononuclear cells of patients with Crohn's disease J Steroid Biochem Mol Biol 2007 103 51 60 10.1016/j.jsbmb.2006.07.008 17049230

[B24] Pang L Nie M Corbett L Knox AJ Cyclooxygenase-2 expression by nonsteroidal anti-inflammatory drugs in human airway smooth muscle cells: role of peroxisome proliferator-activated receptors J Immunol 2003 170 1043 1051 12517972 10.4049/jimmunol.170.2.1043

[B25] Cyrus T Sung S Zhao L Funk CD Tang S Praticò D Effect of low-dose aspirin on vascular inflammation, plaque stability, and atherogenesis in low-density lipoprotein receptor-deficient mice Circulation 2002 106 1282 1287 10.1161/01.CIR.0000027816.54430.96 12208806

[B26] Negrotto S Malaver E Alvarez ME Pacienza N D'Atri LP Pozner RG Gómez RM Schattner M Aspirin and salicylate suppress polymorphonuclear apoptosis delay mediated by proinflammatory stimuli J Pharmacol Exp Ther 2006 319 972 979 10.1124/jpet.106.109389 16936242

[B27] Park HJ Lee SH Son DJ Oh KWan Kim KH Song HS Kim GJ Oh GT Yoon DY Hong JT Antiarthritic effect of bee venom: inhibition of inflammation mediator generation by suppression of NF-kappaB through interaction with the p50 subunit Arthritis Rheum 2004 50 3504 3515 10.1002/art.20626 15529353

[B28] Lee SH Lee SY Son DJ Lee H Yoo HS Song S Oh KW Han DC Kwon BM Hong JT Inhibitory effect of 2'-hydroxycinnamaldehyde on nitric oxide production through inhibition of NF-kappa B activation in RAW 264.7 cells Biochem Pharmacol 2005 69 791 799 10.1016/j.bcp.2004.11.013 15710356

[B29] Park HJ Son DJ Lee CW Choi MS Lee US Song HS Lee JM Hong JT Melittin inhibits inflammatory target gene expression and mediator generation via interaction with IkappaB kinase Biochem Pharmacol 2007 73 237 247 10.1016/j.bcp.2006.09.023 17067557

[B30] Park HJ Lee HJ Choi MS Son DJ Song HS Song MJ Lee JM Han SB Kim Y Hong JT JNK pathway is involved in the inhibition of inflammatory target gene expression and NF-kappaB activation by melittin J Inflamm (Lond) 2008 5 7 10.1186/1476-9255-5-7 18507870 PMC2442592

[B31] Lee KS Kim SR Park HS Park SJ Min KH Lee KY Choe YH Hong SH Han HJ Lee YR Kim JS Atlas D Lee YC A novel thiol compound, N-acetylcysteine amide, attenuates allergic airway disease by regulating activation of NF-kappaB and hypoxia-inducible factor-1alpha Exp Mol Med 2007 39 756 768 18160846 10.1038/emm.2007.82

[B32] Humar M Dohrmann H Stein P Andriopoulos N Goebel U Roesslein M Schmidt R Schwer CI Loop T Geiger KK Pahl HL Pannen BH Thionamides inhibit the transcription factor nuclear factor-kappaB by suppression of Rac1 and inhibitor of kappaB kinase alpha J Pharmacol Exp Ther 2008 324 1037 1044 10.1124/jpet.107.132407 18055877

[B33] Sareila O Hämäläinen M Nissinen E Kankaanranta H Moilanen E Orazipone inhibits activation of inflammatory transcription factors nuclear factor-kappa B and signal transducer and activator of transcription 1 and decreases inducible nitric-oxide synthase expression and nitric oxide production in response to inflammatory stimuli J Pharmacol Exp Ther 2008 324 858 866 10.1124/jpet.107.129114 18039960

[B34] Kwon OC Woo KS Kim TM Kim DJ Hong JT Jeong HS Physicochemical characteristics of garlic (Allium sativum L.) on the high temperature and pressure treatment Korean J Food Sci Technol 2006 38 331 336

[B35] Hwang IG Woo KS Kim DJ Hong JT Hwang BY Lee YR Jeong HS Isolated and identification of an antioxidant substance from heated garlic (Allium sativum L.) Food Sci Biotechnol 2007 16 963 966

[B36] Nath J Powledge A Modulation of human neutrophil inflammatory responses by nitric oxide: studies in unprimed and LPS-primed cells J Leukoc Biol 1997 62 805 816 9400822 10.1002/jlb.62.6.805

[B37] Lowenstein CJ Alley EW Raval R Snowman AM Snyder SH Russel SW Murphy WJ Macrophage nitric oxide synthase gene: two upstream regions mediate induction by interferon γ lipopolysaccharide Proc Natl Acad Sci 1993 90 9730 9734 10.1073/pnas.90.20.9730 7692452 PMC47644

[B38] Lim KM Lee JY Lee SM Bae ON Noh JY Kim EJ Chung SM Chung JH Potent anti-inflammatory effects of two quinolinedione compounds, OQ1 and OQ21, mediated by dual inhibition of inducible NO synthase and cyclooxygenase-2 Br J Pharmacol 2009 156 328 337 10.1111/j.1476-5381.2008.00028.x 19154436 PMC2697840

[B39] Sugishita E Amagaya S Ogihara Y Anti-inflammatory testing methods: comparative evaluation of mice and rats J Pharmacobiodyn 1981 4 565 575 7299620 10.1248/bpb1978.4.565

[B40] Geller DA Lowenstein CJ Shapiro RA Nussler AK Kisilvio M Wang SC Nakayama DK Simmons RL Snyder SH Billiar TR Molecular cloning and expression of inducible nitric oxide synthase from human hepatocytes Proc Natl Acad Sci 1993 90 3491 3495 10.1073/pnas.90.8.3491 7682706 PMC46326

[B41] Sastre M Klockgether T Heneka MT Contribution of inflammatory processes to Alzheimer's disease: molecular mechanisms Int J Dev Neurosci 2006 24 167 176 10.1016/j.ijdevneu.2005.11.014 16472958

[B42] Korhonen R Lahti A Kankaanranta H Moilanen E Nitric oxide production and signaling in inflammation Curr Drug Targets Inflamm Allergy 2005 4 471 179 10.2174/1568010054526359 16101524

[B43] Naseem KM The role of nitric oxide in cardiovascular diseases Mol Aspects Med 2005 26 33 65 10.1016/j.mam.2004.09.003 15722114

[B44] Suschek CV Schnorr O Kolb-Bachofen V The role of iNOS in chronic inflammatory processes in vivo: is it damage-promoting, protective, or active at all? Curr Mol Med 2004 4 763 775 10.2174/1566524043359908 15579023

[B45] Tsatsanis C Androulidaki A Venihaki M Margioris AN Signalling networks regulating cyclooxygenase-2 Int J Biochem Cell Biol 2006 38 1654 1661 10.1016/j.biocel.2006.03.021 16713323

[B46] Dijkstra G Moshage H Jansen PL Blockade of NF-kappaB activation and donation of nitric oxide: new treatment options in inflammatory bowel disease? Scand J Gastroenterol Suppl 2002 236 37 41 10.1080/003655202320621436 12408502

[B47] Surh YJ Chun KS Cha HH Han SS Keum YS Park KK Lee SS Molecular mechanisms underlying chemopreventive activities of anti-inflammatory phytochemicals: down-regulation of COX-2 and iNOS through suppression of NF-kappa B activation Mutat Res 2001 480-481 243 268 11506818 10.1016/s0027-5107(01)00183-x

[B48] De Stefano D Maiuri MC Iovine B Ialenti A Bevilacqua MA Carnuccio R The role of NF-kappaB, IRF-1, and STAT-1alpha transcription factors in the iNOS gene induction by gliadin and IFN-gamma in RAW 264.7 macrophages J Mol Med 2006 84 65 74 10.1007/s00109-005-0713-x 16284791

[B49] Simmonds RE Foxwell BM Signalling, inflammation and arthritis: NF-kappaB and its relevance to arthritis and inflammation Rheumatology 2008 47 584 590 10.1093/rheumatology/kem298 18234712

[B50] Cao S Zhang X Edwards JP Mosser DM NF-kappaB1 (p50) homodimers differentially regulate pro- and anti-inflammatory cytokines in macrophages J Biol Chem 2006 281 26041 26050 10.1074/jbc.M602222200 16835236 PMC2642587

[B51] Kawano S Kubota T Monden Y Tsutsumi K Inoue T Kawamura N Tsutsui H Sunagawa K Blockade of NF-kappaB improves cardiac function and survival after myocardial infarction Am J Physiol Heart Circ Physiol 2006 291 H1337 1344 10.1152/ajpheart.01175.2005 16632551

[B52] Kanters E Gijbels MJ Made I van der Vergouwe MN Heeringa P Kraal G Hofker MH de Winther MP Hematopoietic NF-kappaB1 deficiency results in small atherosclerotic lesions with an inflammatory phenotype Blood 2004 103 934 940 10.1182/blood-2003-05-1450 14512319

[B53] Natarajan K Singh S Burke TR Jr Grunberger D Aggarwal BB Caffeic acid phenethyl ester is a potent and specific inhibitor of activation of nuclear transcription factor NF-kappa B Proc Natl Acad Sci USA 1996 93 9090 9095 10.1073/pnas.93.17.9090 8799159 PMC38600

[B54] García-Piñeres AJ Castro V Mora G Schmidt TJ Strunck E Pahl HL Merfort I Cysteine 38 in p65/NF-kappaB plays a crucial role in DNA binding inhibition by sesquiterpene lactones J Biol Chem 2001 276 39713 39720 10.1074/jbc.M101985200 11500489

[B55] Son DJ Park MH Chae SJ Moon SO Lee JW Song HS DC Moon Kang SS Kwon YE Hong JT Inhibitory effect of snake venom toxin from Vipera lebetina turanica on hormone-refractory human prostate cancer cell growth: induction of apoptosis through inactivation of nuclear factor kappaB Mol Cancer Ther 2007 6 675 683 10.1158/1535-7163.MCT-06-0328 17308063

